# Soft Tissue Augmentation with Autologous Platelet Gel and *β*-TCP: A Histologic and Histometric Study in Mice

**DOI:** 10.1155/2016/2078104

**Published:** 2016-07-12

**Authors:** Antonio Scarano, Maurizio Ceccarelli, Massimiliano Marchetti, Adriano Piattelli, Carmen Mortellaro

**Affiliations:** ^1^Department of Medical, Oral and Biotechnological Sciences and CeSI-MeT, University of Chieti-Pescara, Via dei Vestini 31, 66100 Chieti, Italy; ^2^Department of Medical, Oral and Biotechnological Sciences, University of Chieti-Pescara, Via dei Vestini 31, 66100 Chieti, Italy; ^3^Department of Surgery Sciences, University of Rome “La Sapienza”, Piazzale Aldo Moro 5, 00185 Roma, Italy; ^4^Oral Surgery Unit, Regina Margherita Pediatric Hospital and University of Eastern Piedmont, Viale Piazza d'Armi 1, 28100 Novara, Italy

## Abstract

*Background*. Facial aging is a dynamic process involving both soft tissue and bony structures. Skin atrophy, with loss of tone, elasticity, and distribution of facial fat, coupled with gravity and muscle activity, leads to wrinkling and folds.* Purpose*. The aim of the study was to evaluate microporous tricalcium phosphate (*β*-TCP) and autologous platelet gel (APG) mix in mice for oral and maxillofacial soft tissue augmentation. The hypothesis was that *β*-TCP added with APG was able to increase the biostimulating effect on fibroblasts and quicken resorption.* Materials and Methods*. Ten female, 6–8-week-old black-haired mice were selected. *β*-TCP/APG gel was injected into one cheek; the other was used as control. The animals were sacrificed at 8 weeks and histologically evaluated.* Results*. The new fibroblast was intensively stained with acid fuchsin and presented in contact with *β*-TCP. At higher magnification, actively secreting fibroblasts were observed at the periphery of *β*-TCP with a well differentiated fibroblast cell line and blood vessels. Acid fuchsin stained cutaneous structures in pink: no epidermal/dermal alterations or pathological inflammatory infiltrates were detected. The margins of *β*-TCP granules were clear and not diffused near tissues.* Conclusion*. APG with *β*-TCP preserves skin morphology, without immune response, with an excellent tolerability and is a promising scaffold for cells and biomaterial for soft tissue augmentation.

## 1. Introduction

Facial aging is characterized by skin changes, sagging, and volume loss. Vashi et al. detected that this process begins with surface and subsurface structural changes in multiple facial tissue layers, including skin, fat, muscle, and bone [1]. Facial tissue layers age interdependently, contributing to the overall facial appearance. As discussed by Richard et al. skeletal bone is a scaffold for the soft tissue [2]. Facial aging is a dynamic process involving the aging of soft tissue and bony structures. Sjerobabski-Masnec and Šitum reported that a multisystem degenerative process involves the skin and the skin support systems including the bone, cartilage, and subcutaneous compartments [3].

Recent evidence from Mendelson et al. clearly demonstrates that the aging of the face is primarily one of bone resorption [4] and changes in bones and the facial skeleton have a significant effect [5]. So, as seen by Levine et al., any kind of changes in the mandibular and maxilla projection, width or height, can affect overall aesthetics [6]. The midface skeleton is formed by the maxilla in the medial and middle thirds and, as highlighted by Soares and Silver, it is completed by the body and arch of the zygoma in the lateral third [7]. The maxillary angle (superior-to-inferior maxilla at the articulation of the inferior maxillary wing and alveolar arch) of young and old patients changes significantly with bone resorption and loss of projection of the maxilla. Such patients often benefit from volumetric augmentation to enhance their skeletal support.

Evian et al. reported that several techniques for managing such deformities have been proposed, either by soft or hard tissue augmentation [4–8]. As seen by André, soft tissue augmentation, with absorbable injectable filling agents in the facial region, is rapidly increasing in popularity [9]. In addition, the proven safety of these products substantially contributes to their widespread use. The filling of tissue for facial rejuvenation can be challenging. The aim is to restore harmonious volumetric facial ratios by regaining the contours of the cheekbones, drooping jaw lines and nasolabial folds. As previously reported, soft tissue augmentation is frequently required for the reconstruction of perioral skin tissues in facial deformities, trauma, or for intraoral ridge defects [1, 2], causing functional problems and/or aesthetic complaints.

Dermal fillers are commonly subdivided on the basis of their turnover in the skin: temporarily, semipermanent (duration is often longer than 18 months, but the exact time frame has not been established), and permanent with a wide range of choices as well as specific indications. The scientific opinion for the application of a substance for soft tissue augmentation is critical.

One of the core concepts of soft tissue augmentation is the three-dimensional improvement in appearance. A youthful face has a soft, full appearance, as opposed to the flat, pulled, bidimensional tending face skin often achieved by more traditional surgical approaches. Injectable filling agents can augment and even, at times, replace pulling. These techniques produce a volumetric increase of modest dimensions for a short time. The hypothesis was that tricalcium phosphate mixed with APG had a slow resorption and a biostimulating effect on fibroblasts.

Scarano et al. reports that* microporous* tricalcium with autologous platelet-derived growth factors has been proposed to increase the volume and the duration of soft tissue augmentation [10].

The aim of the present study was the histological evaluation of* microporous* tricalcium phosphate mixed with autologous platelet gel injected subdermally below the cheek regions of mice.

## 2. Materials and Methods

### 2.1. Subject Animal

The black-haired mice used in this study were obtained from the laboratory of University of Chieti-Pescara, Italy. Ten female, 6–8-week-old black-haired mice were selected. The procedures were approved by Interinstitutional Ethics Committee for Animal Experimentation of University of Chieti-Pescara, Italy (prot. 06/2012/CEISA/PROG31), and this study was performed according to the European Community guidelines (ED 2010/63/UE).

All animals were anesthetized by an intramuscular injection (average dose, 8 mL/kg) of a mixture of ketaminium hydrochloridum and xylazine hydrochloridum diluted with saline (2.1 mL of 50 mg/mL Ketalar (Parke-Davis, Zaventem, Belgium), 0.3 mL of 2% Rompun (Bayer, Leverkusen, Germany), and 3.4 mL of 0.9% NaCl). This protocol provided good anesthesia during the infiltration period of the autologous platelet-derived growth factor and *β*-tricalcium phosphate.

### 2.2. Test Procedure

This study evaluated* in vivo* a micro *β*-tricalcium phosphate Ca_3_(PO_4_)_2_ (Skin-hyxa, Italfarmacia srl, Rome, Italy) architecture with 20–30 *μ*m mixed with the autologous platelet-derived growth factor. Explants harvested after 8 weeks were histologically examined for the evaluation of soft tissue augmentation in mice.

Approximately 0.1 cc of blood was collected into collection tubes via standard venipuncture, using a 27-gauge butterfly needle. The sample was agitated to thoroughly mix the anticoagulant with the blood. Then the tubes with sodium citrate 3.8% were immediately centrifuged at 480 RCF for 4 min with centrifuge (GF One, UBGEN, Padova, Italy). Following centrifugation, the blood sample was separated in different blood fractions: the red blood cells formed a red-colored fraction on the bottom of the tube that was separated by the buffy coat and a whitish layer rich in white blood cells and autologous platelet gel (APG), which contained autologous fibrinogen and was poor in platelets.

**Figure 1:**
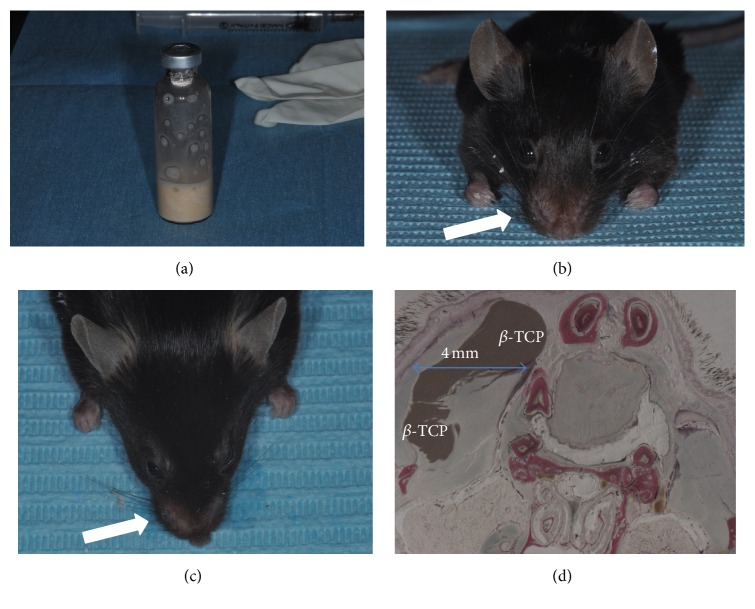
(a) Preparing the gel of tricalcium phosphate/autologous platelet-derived growth factors. (b) Injecting the autologous platelet gel growth factors mixed with *β*-TCP produces a soft tissue augmentation (arrow). (c) After 8 weeks the increase of the cheek is still present. (d) The *β*-TCP is distributed between the bone and dermal tissues and not diffused within the vicinity of the tissues. Acid fuchsin and toluidine blue 2x.

According to the protocol the syringe was used to aspirate the layer rich in platelets. Approximately 0.02 cc of APG was produced per tube. This activated suspension was mixed together with tricalcium phosphate. We mixed 20% APG to *β*-TCP for easier application of the tricalcium phosphate due to the liquefaction effect of platelet-derived growth factor (Figure 1(a)). The total preparation time from venipuncture to injection was approximately 8 min.

The mixture was then subdermally injected below the cheek regions through 22-gauge needles, to achieve the most soft tissue augmentation for an optimal correction, with mainly a bolus technique (Figure 1(b)). 0.1 cc gel of tricalcium phosphate/autologous platelet gel was injected into one cheek, while the other cheek was left empty and was used as control. Total treatment time (from venipuncture to completion of treatment) was less than 10 min in most cases. After procedure the mice were maintained in a controlled environment at a temperature of 24°C, a relative humidity of 55%, and a 12 h light cycle. The animals were sacrificed, with an overdose of intravenous pentobarbital after 8 weeks.

### 2.3. Histomorphometry

The specimens were washed in saline solution and immediately fixed in 4% paraformaldehyde and 0.1% glutaraldehyde in 0.15 M cacodylate buffer at 4°C and pH 7.4, for histological processing. Thin ground sections were obtained with the Precise 2 Automated System (Assing, Roma, Italy). The specimens were dehydrated in an ascending series of alcohol rinses and embedded in a glycol methacrylate resin (Technovit 7200 VLC, Kulzer, Wehrheim, Germany).

After polymerization the specimens were sectioned in the craniocaudal direction, with a high-precision diamond disc at approx. 150 *μ*m and ground down to approx. 30 *μ*m with a specially designed grinding machine. Two slides for each site were prepared for a total of 10 tests and 10 controls. The slides were stained with acid fuchsin and toluidine blue and they were observed in normal transmitted light under a Leitz Laborlux microscope (Leitz, Wetzlar, Germany).

Histomorphometry was carried out using a light microscope (Laborlux S, Leitz, Wetzlar, Germany) connected to a high resolution video camera (3CCD, JVC KY-F55B, JVC®, Santa Clara, CA, USA) and interfaced to a monitor and PC (Intel Pentium III 1200 MMX, Intel®, Yokohama, Japan). This optical system was associated with a digitizing pad (Matrix Vision GmbH, Oppenweiler, Germany) and a histometry software package with image capturing capabilities (Image-Pro Plus 4.5, Media Cybernetics Inc., Immagini & Computer Snc Milano, Italy).

The tissue thickness between the bone and skin epithelium of the two experimental groups was evaluated. Each section was examined at a minimum of 2x magnification, and the entire area of the section was evaluated. Three different readings for each slide were performed and an image manipulation software was used to create individual tissue thickness and fibroblast for 1 × 1 mm field. These layers were then converted to a binary (black and white) form, and area by percentage of each of the three layers based on the number of pixels was digitally calculated using image analysis software.

### 2.4. Statistical Analysis

A power analysis was performed using clinical software, freely available on the site http://clincalc.com/stats/samplesize.aspx, for determining the number of animals needed to achieve statistical significance for quantitative analyses of histomorphometry. A calculation model was adopted for dichotomous variables (yes/no effect) by putting the effect incidence designed to caution the reasons as 10% for controls and 95% for treated. The optimal number of samples for analysis is 10 mice.

Differences between groups of treatment were analyzed by one-way analysis of variance (ANOVA) followed by Fisher's Protected Least Significant Difference (PLSD) post hoc test. A *p* value <0.05 was considered statistically significant. Statistical analysis was performed using the Statview software from SAS Institute.

## 3. Results

**Figure 2:**
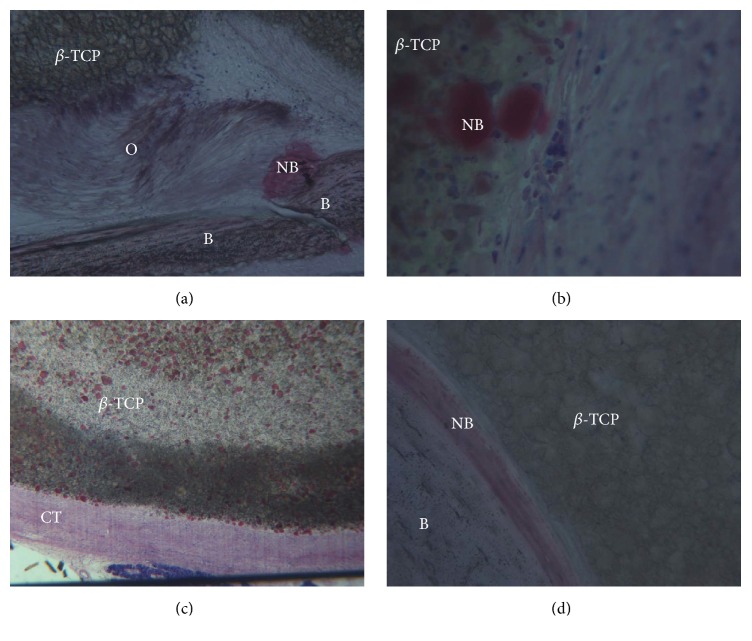
(a) *β*-TCP particles surrounded by osteoid matrix and new bone. Acid fuchsin and toluidine blue 100x. (b) An initial formation of immature bone (NB) extending from the periphery of the *β*-TCP is present. Lymphocytes were present around the *β*-TCP. Acid fuchsin and toluidine blue 200x. (c) *β*-TCP is completely surrounded by connective tissue (CT). No inflammatory cell infiltrate or macrophages are present. Acid fuchsin and toluidine blue 50x. (d) Only around some particles is it possible to observe the presence of new bone (NB) and mature bone. Acid fuchsin and toluidine blue 250x.

The micro *β*-tricalcium phosphate (*β*-TCP) was surrounded by soft tissues (Figures 1(c) and 1(d)). The new fibroblasts appeared intensively stained with acid fuchsin and presented in contact with *β*-TCP (Figures 2(a) and 2(b)). At higher magnification, many actively secreting fibroblasts were observed in the periphery of the *β*-TCP. There were well differentiated fibroblast cell lines and blood vessels. Acid fuchsin stained cutaneous structures in pink. The skin structure was intact: neither epidermal or dermal alterations nor pathological inflammatory infiltrates were detected 8 weeks after the injection. Furthermore, when toluidine blue staining was applied, only very few mast cells were observed in the skin sections.

**Figure 3:**
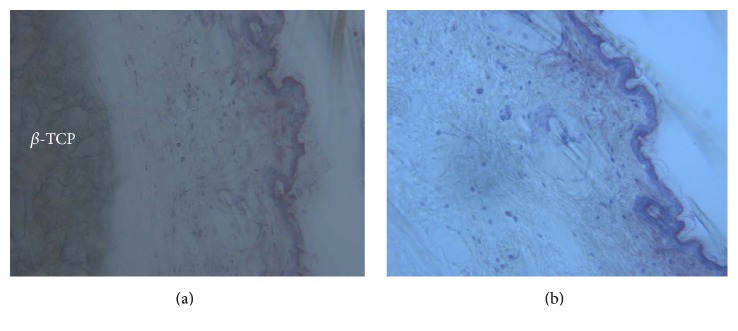
(a) Test site. No granuloma is detected. The skin structure is intact: no epidermal or dermal alterations nor pathological inflammatory infiltrates are detected. Acid fuchsin and toluidine blue 100x. (b) Control site. The skin structure is similar to the test site. Acid fuchsin and toluidine blue 100x.

The *β*-TCP was distributed between the bone and dermal tissues (Figures 1(c) and 1(d)). The *β*-TCP formed large aggregates distributed irregularly throughout the entire cross section of the reticular dermis. The spreading of the *β*-TCP was more restricted in the vertical axis, and thus it did not reach the proximity of the papillary dermis. No induction of myofibroblasts was observed. No granuloma was detected and there were a few isolated macrophages randomly distributed in the dermis (Figures 2(c) and 2(d)). Traces of new bone from the periosteum were observed, overlying the superficial portion of the bone *β*-TCP (Figures 2(a), 2(b), and 2(d)). Acid fuchsin staining revealed that cutaneous structures were morphologically intact and no inflammation was induced by the injection of all the tested fillers (Figure 3(a)).

Areas of resorption were present on the surface of some graft particles. No inflammatory or foreign body cells were present. In some areas small capillaries were present between the particles. Histomorphometry showed that tissue thickness was 4 ± 0.6 mm, and the number of fibroblast per field was 57 ± 7.

*In the control group*, acid fuchsin staining revealed that cutaneous structures were morphologically intact and no inflammation was present (Figure 3(b)). The presence of only a few macrophages in all the analyzed skin sections was observed. In all specimens no pathologically inflammatory cell infiltrate was present. No foreign body reactions were present at any time. In some areas small capillaries were present between the particles. Acid fuchsin stained cutaneous structures in pink (Figure 3(b)). It can be seen that the skin structure was intact: no epidermal or dermal alterations or pathological inflammatory infiltrates were detected. A well differentiated fibroblast cell line and blood vessels were observed. Furthermore, when toluidine blue staining was applied, only very few mast cells were observed in the skin. Histomorphometry showed that tissue thickness was 1.5 ± 0.2 mm, and the number of fibroblast per field was 37 ± 4.

### 3.1. Statistical Analysis

**Figure 4:**
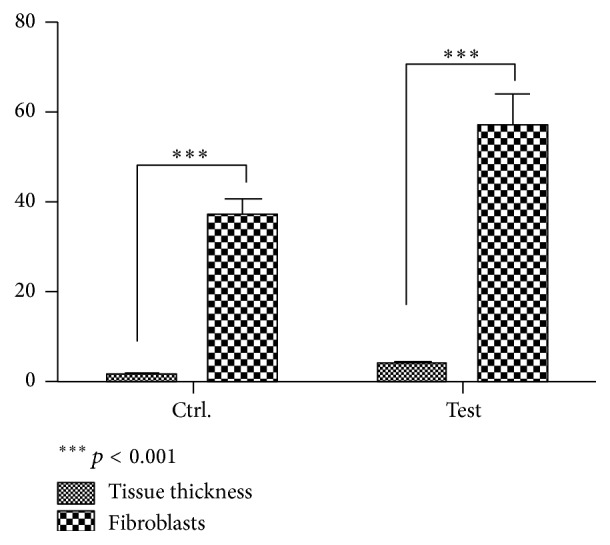
Higher and highly statistically significant differences were found in the tissue thickness and the number of fibroblasts per field in the control group versus the test group.

All experimental groups showed an increase of tissue thickness. Higher and highly statistically significant differences were found in the tissue thickness and the number of fibroblast per field in the control group versus the test group (Figure 4).

## 4. Discussion

Over the past two decades, the treatment of soft tissue loss (congenital, acquired, or senile) with dermal filler has shown encouraging clinical results in terms of texture, softness, and quality of skin pattern.

Hammond et al. highlighted the fact that autologous platelets have been used over the last several years as an effective treatment in various surgical and medical fields [11, 12]. According to the literature, many protocols exist. The general principle of production consists of centrifugation, making it possible to eliminate red blood cells, and then acellular plasma, to preserve only the concentrated platelets. Hamilton et al. reported that autologous platelet-derived growth factors have been used over the last several years as an effective treatment in various surgical and medical fields [13, 14]. Platelets are an excellent source of GFs in their naturally occurring and biologically determined ratio and are successful in acute wound healing.

According to Scimeca et al. the application of autologous platelet-derived growth factors has been proven to enhance early wound healing and healing in diabetic ulcers [15]. Concentrated platelet preparations have been used clinically since the 1990s to simulate a native wound healing environment and then compare with it after isolated growth factor application.

There is also substantial clinical proof of autologous platelet-derived growth factors in other areas of medicine. For example, as confirmed by Fortier et al., autologous platelet gel is widely used in orthopedics and oral maxillofacial surgery [16, 17]. Hom et al. described that these growth factors include vascular endothelial growth factor (VEGF), platelet-derived growth factor (PDGF), epidermal growth factor (EGF), insulin-like growth factor-1 (IGF-1), basic fibroblast growth factor (bFGF), transforming growth factor-b1 (TGF-b1), transforming growth factor a (TGF-a), platelet activating factor (PAF), thrombospondin, platelet thromboplastin, coagulation (coagulascion) factors, serotonin, histamine, hydrolytic enzymes, and endostatin [18]. Ceccarelli and García proposed the use of platelet growth factors in dermal biostimulation [19] and subsequently Scarano et al., as discussed elsewhere, proposed the use of a mix of *β*-TCP for soft tissue augmentation.

Tricalcium phosphate (*β*-TCP) is a rather new material in aesthetic medicine as it is dispersed in hyaluronic acid. We have used the *β*-TCP mixed with APG for a stable preparation for injecting. When injected into subcutaneous tissues it produces a mechanism of insult and lesion with the production of a new collagen matrix. This insult is an extraneous body and occurs with tricalcium phosphate: a mechanism of reaction and reparation that gives results. The reparation reaction is probably stimulated by APG. This synergistic effect increases the presence of fibroblasts and the production of collagen.

The material is composed of microparticles of synthetic ceramic, biocompatible, biodegradable, and immunologically inert, with a diameter of 20–30 microns. Probably the *β*-TCP absorbs the growth factor present in the APG and slowly releases them. The growth factor is released in specific ratios and works in concert and in a specific order to attract inflammatory cells and fibroblasts, as well as to stimulate collagen deposition and endothelial budding. These features lead to appropriate wound healing.

Obviously, what was originally designed for topical application is now under review in the light of introduction by injection into the dermis. After injection into the dermis and subcutaneous layers, the platelets are activated endogenously by the body's own coagulation factors such as thrombin and collagen. This leads to platelet degranulation, releasing platelet GFs such as PGDF, ILGF, EGF, and *β*-TGF. Activated platelets also release proteins such as the adhesive glycoproteins, fibrin, fibronectin, and vitronectin. These proteins and GFs interact with cells in the subcutaneous tissues, such as fibroblasts, endothelial cells, and stem cells. After binding to their cellular receptors, they activate intracellular signaling events mediating cell proliferation, migration, survival, and production of extracellular matrix proteins. As seen by Scarano et al. this results in tissue augmentation [20]. While *β*-TGF volume increases for a mild reaction with a formation of fibrotic collagen, this biostimulation is safe and creates an immediate and long-lasting volumetric effect and a natural result.

In this study we observed a new osteogenesis near the native bone and this evidence was very interesting. In fact, in literature, it is demonstrated that the aging of the face is the result of bone resorption [4] and changes of the facial skeleton. The bone formation observed in this study could be seen as a positive evidence that* in vivo* soft tissues are naturally supported by the skeletal tissue. In fact the bone formation is present near the native bone, but not in the soft tissues. Face rejuvenation with autologous platelet-derived growth factors is a promising, easy technique, performing favorably in all small skin wrinkles, as well as in skin texture and elasticity. It is considered a long-lasting, but nonpermanent filler and is highly biocompatible. The results indicate that micro *β*-tricalcium phosphate mixed with autologous platelet gel material is able to create a lasting three-dimensional soft tissue augmentation and is a promising biomaterial for soft tissue augmentation as a scaffold for cells.

Considering the high demand for soft tissue augmentation fillers that exist on the market, safety remains a paramount requirement for all products. Our results show that the APG with *β*-TCP preserves skin morphology, does not induce immune response, and therefore has excellent tolerability.
